# Dielectric spectroscopy of isotropic liquids and liquid crystal phases with dispersed graphene oxide

**DOI:** 10.1038/srep31885

**Published:** 2016-08-24

**Authors:** Shakhawan Al-Zangana, Maria Iliut, Gökçen Boran, Michael Turner, Aravind Vijayaraghavan, Ingo Dierking

**Affiliations:** 1 School of Physics and Astronomy, University of Manchester, Oxford Road, Manchester M13 9PL, United Kingdom; 2 School of Materials and National Graphene Institute, University of Manchester, Oxford Road, Manchester M13 9PL, United Kingdom; 3 Physics Department, Bogazici University, Istanbul, 34342, Turkey; 4 School of Chemistry, University of Manchester, Oxford Road, Manchester M13 9PL, United Kingdom

## Abstract

Graphene oxide (GO) flakes of different sizes were prepared and dispersed in isotropic and nematic (anisotropic) fluid media. The dielectric relaxation behaviour of GO-dispersions was examined for a wide temperature (25–60 ^o^C) and frequency range (100 Hz–2 MHz). The mixtures containing GO flakes exhibited varying dielectric relaxation processes, depending on the size of the flakes and the elastic properties of the dispersant fluid. Relaxation frequencies of the GO doped isotropic media, such as isopropanol IPA, were observed to be much lower than the GO doped thermotropic nematic medium 5CB. It is anticipated that the slow relaxation frequencies (~10 kHz) could be resulting from the relaxation modes of the GO flakes while the fast relaxation frequencies (~100 kHz) could indicate strongly slowed down molecular modes of the nematogenic molecules, which are anchored to the GO flakes via dispersion interactions. The relaxation frequencies decreased as the size of the GO flakes in the isotropic solvent was increased. Polarizing microscopy showed that GO flakes with a mean diameter of 10 μm, dispersed in water, formed a lyotropic nematic liquid crystal phase. This lyotropic nematic exhibited the slowest dielectric relaxation process, with relaxation frequencies in the order of 2 kHz, as compared to the GO-isotropic suspension and the GO-doped 5CB.

Due to their outstanding physical and chemical features, graphene research and that of other two-dimensional materials, including oxides, have recently exhibited much interest^1^,^2^,^3^,^4^. This was only made possible when the challenge to produce monolayer graphene was solved through mechanical exfoliation by Novoselov *et al*.^5^. Graphene oxide (GO) is a one- or very few-atomic-layer thick material, produced by the mechanical exfoliation of graphite oxide, yielding sheets decorated with hydroxyl and epoxide functional groups on the surface and carbonyl and carboxyl groups at the edges^6^,^7^. This makes the electrical conductivity of GO much smaller as compared to that of graphene^8^ and provides a very high dielectric permitivity^9^.

Due to the hydrophilic nature of GO, molecules of polar solvents easily intercalate into the GO layers^10^. The self-assembly of GO flakes in isotropic media (often water) has been found to result in a lyotropic nematic liquid crystal when the concentration of the GO exceeded approximately 1 mg/mL^6^,^7^,^11^,^12^,^13^. The formation of this phase depends on the polarity of the medium and the size of the GO flakes. Due to its high polarity, water has been shown to be an ideal solvent for the formation of stable GO dispersions and facilitating lyotropic nematic mesomorphism. On the other hand, the electro-optic response of a thermotropic nematic liquid crystal has been shown to be improved through doping with GO^14^. The coupling of any electrical dipoles on the surface of the GO flakes with the electrical dipoles of the mesogen and the trapping of the always present ionic contamination by GO flakes could be the main reason behind the dielectric gain and the improved electro-optic behaviour of liquid crystal-GO dispersions.

The nematic is the most common and the simplest of the liquid crystalline phases, the one with the highest symmetry. Molecules possess long-range orientational order, but no positional order of their centres of mass, with the long axis of the molecules aligning roughly along an average direction, called the director ***n***^15^. The common uniaxial nematic phase has two characteristic molecular dielectric relaxation processes; the high frequency relaxation (~10^9^ Hz), which arises from the rotation of the molecules around the long molecular axis, and the lower frequency relaxation (~10^7^ Hz), which stems from the rotation around the short molecular axis^16^,^17^.

In this paper, we investigate the dielectric behaviour of different average size ranges of the GO microparticles suspended in isotropic solvents, as well as a standard thermotropic nematic liquid crystal, 5CB. IPA is utilized as a solvent for the GO flakes whose average size is not large enough to exhibit a lyotropic nematic phase. Larger GO flakes, which show the lyotropic nematic phase, are dispersed in deionized water. We present systematic dielectric spectroscopy results of GO in different phases, at varying temperature and for different GO sizes.

## Material and Method

### Sample preparation

Graphene oxide was first prepared from graphite flakes and dispersed in water by the modified Hummers method^18^,^19^. The dispersions of graphene oxide in the thermotropic nematic liquid crystal, 5CB, were prepared following the procedure described in ref. 14. The graphene oxide flakes in water are transferred by solvent exchange to IPA, with a known concentration. Mixtures of different weight percentage (wt.%) of GO flakes were prepared by adding a definite amount of GO-IPA to 70 mg of 4-Cyano-4′-pentylbiphenyl (5CB, from SYNTHON Chemicals GmbH & Co. KG, Germany).

The dielectric measuring cells were home-built from ITO-coated glass (VisionTek Systems Ltd.) of thickness 1.1 mm, and ITO resistance of 10 Ω/◽. The glass was cut into 1.5 cm × 2 cm substrates and then washed in organic solvents (acetone and methanol) in a sonication bath for 30 mins. The ITO was etched leaving the central parts of the glass with 0.5 cm width of ITO and removing the ITO on the two opposite edges. The etching was performed by using Kapton tape as a protective layer and immersing the glass in hydrochloric acid (HCl-30%) for 7 mins. The substrates were again washed, dried and treated in a plasma chamber for 2.5 minutes (Pico Plasma System) to remove any organic residues. For the GO-5CB samples the substrates were then spin-coated with a solution of PVA-water (0.5 mg/ml), and unidirectionally, antiparallel rubbed with a velvet cloth to achieve homogeneous planar alignment. The GO + 5CB mixture was sandwiched between the ITO glasses to avoid GO size selection at the cell opening, and was then sealed with UV glue (Norland N68). The cell thickness was controlled at the edges where the ITO layer was previously removed, by a Mylar spacer of thickness 13 μm. The sandwich cells for the GO-IPA mixtures were prepared with the same technique but without alignment layer. The GO flakes in the isotropic solvents IPA and water of concentrations 0.4 mg/mL were left for two weeks to settle. The bottom layer with highly concentrated GO was used to fill the dielectric cells by capillary action.

### Experimental technique

The frequency-dependent capacitance C and the dissipation factor (loss tangent) tanδ of the cells were measured using an Agilent Precision LCR Meter E4980A, which was operated in the parallel equivalent circuit in the frequency range of 20 Hz to 2 MHz at a measuring voltage V_ac_  = 0.1 V, well below the threshold voltage of the liquid crystal. The storage component (real part) ε′ and the loss component (imaginary part) ε” of the complex dielectric permittivity are then calculated according to the following equations:


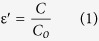


where C_o_ is the capacitance of the empty cell, and C is the capacitance when the cell is filled with the liquid crystal and


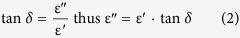


Texture acquisition was carried out by means of a Leica DMLP polarizing microscope and a uEye digital camera at resolution 2048 × 1088 pixels. A temperature controller (Linkam TMS 94) with relative accuracy ±0.1 K controlled the sample temperature through the hot-stage chamber. Experiments and data acquisition were automated using LabView^TM^.

For the data analysis the Havriliak-Negami (HN) equation was used to fit the relaxation modes as a function of frequency, f, of the applied electric measuring field [2]:


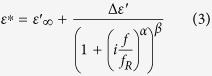


where ε^*^ is the complex dielectric permittivity, 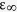 is the dielectric permittivity at high frequencies. 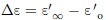 is the dielectric strength, with 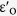 the dielectric permittivity at low-frequency, *f*_*R*_ is the relaxation frequency, *α* a fitting parameter which describes the width of the loss peak, and *β* a further fitting parameter which accounts for a possible asymmetry of the loss peak.

In the Debye relaxation model^20^ with a single relaxation frequency it is *α* = *β* = 1, the Cole-Cole model^21^,^22^ with a symmetric distribution of the relaxation frequency has 0 ≤ *α* ≤ 1 and *β* = 1, and the Cole-Davidson model^23^,^24^ with an asymmetric distribution of the relaxation peak relies on *α* = 1 and 0 ≤ *β* ≤ 1. the most general case is described by the Havriliak-Negami model with 0 ≤ *α* ≤ 1 and 0 ≤ *β* ≤ 1. The real and imaginary parts of the complex dielectric constant are related to each other by the Kramers–Kronig relation^25^:


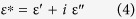


**Figure 1:**
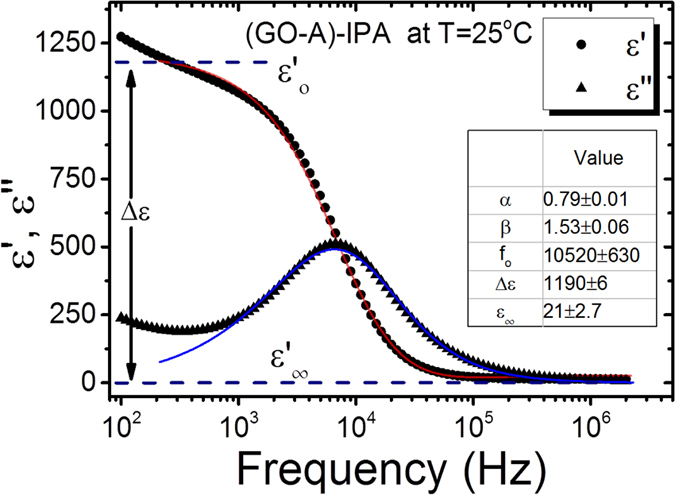
Example of the simultaneous fitting process of the complex dielectric permittivity using the Havriliak-Negami equation, here for GO-A flakes dispersed in IPA (see below). The solid lines represent fitted data. Obtained parameters are indicated in the inset.

The frequency dependence of ε′ and 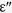 thus has to be fitted simultaneously to obtain the dielectric strength and the relaxation frequency, besides the above mentioned further parameters. An exemplary simultaneous fit is depicted in Fig. 1.

### Characterization of the GO flakes

**Figure 2:**
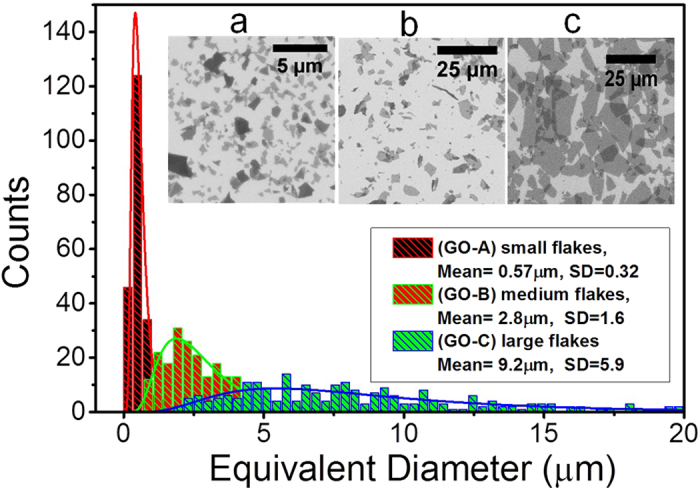
Scanning electron microscopy (SEM) images of the GO flakes. (**a**) GO-A, (**b**) GO-B and (**c**) GO-C. The size (equivalent diameter) distribution of GO flakes obtained by SEM, the mean size, and its standard deviation SD for all flake batches are indicated in the inset, (note the difference in scale in the SEM images).

The size distribution, average size, and polydispersity of GO flakes for all the three flake types were characterized using scanning electron microscopy (SEM), which is demonstrated in (Fig. 2). The GO flakes consisted of irregular polygonal shapes with relatively wide size distributions. Flake batches abbreviated as (GO-A), (GO-B) and (GO-C) were found to have mean equivalent diameters of (0.57 ± 0.32) μm, (2.8 ± 1.6) μm, and (9.2 ± 5.9) μm, respectively. As shown in the inset of (Fig. 2), the size distribution covers smaller fragments, which are thought to be produced as a result of breaking down the larger flakes during the exfoliation process^6^. A similar characterization of the size distribution of graphene oxide flakes has also been reported for photoluminescence imaging^26^.

## Results and Discussion

### Optical Polarizing microscope textures

**Figure 3:**
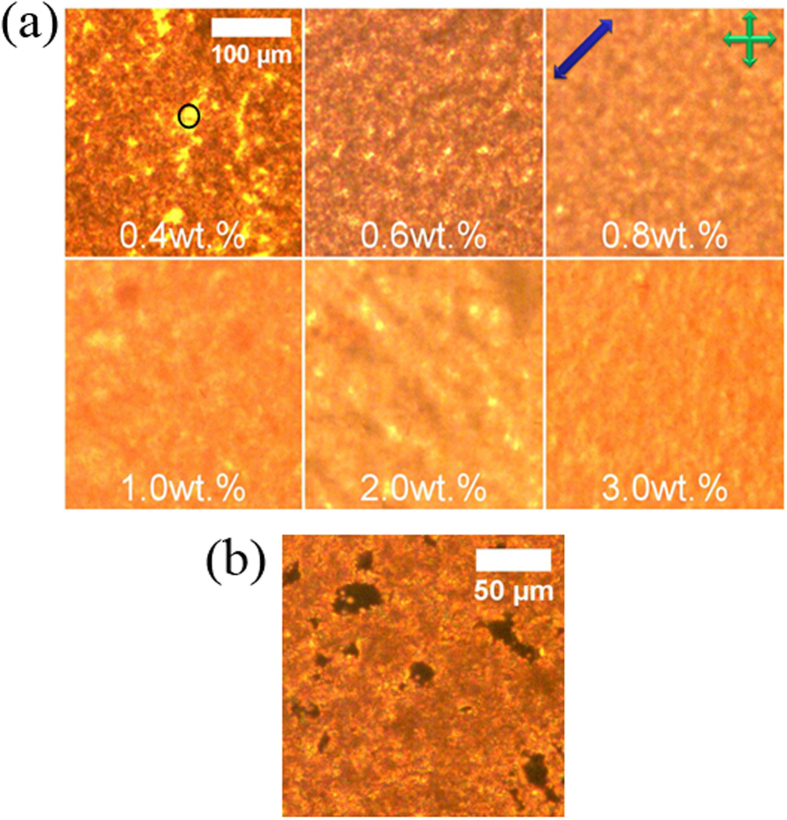
(**a**) Polarizing optical images obtained from liquid crystal cells with planar boundary conditions, filled with (GO-A) + 5CB mixtures at concentrations of 0.4, 0.6, 0.8, 1.0, 2.0 and 3.0 wt.%. Bright areas (inside the circle) indicate an undisturbed liquid crystal director orientation while darker areas show graphene oxide aggregates. The rubbing direction (blue arrow) is aligned at 45° to the crossed polarizers (crossed green arrows). (b) The bottom image is for a GO-A + 5CB filled cell at concentration 0.4 wt.% with the director parallel to one of the polarizers. One can clearly see remains of liquid crystal regions (black), surrounded by graphene oxide aggregates with a highly distorted director field. An equivalent behaviour is observed for the other samples.

Polarizing microscopic images of the mixtures of (GO-A) in 5CB are shown in Fig. 3, as a function of increasing GO concentration. Only samples of relatively high doping concentrations are shown because dielectric relaxation processes have only been observed for concentrations above 0.4 wt.%. As shown in Fig. 3, the area appearing with orange colour inside the indicative circle, exhibits homogeneous alignment with the director being undisturbed by dispersed flakes. On the other hand, the dark brown areas outside the circle illustrate a GO aggregate, which disturbs the liquid crystal alignment. The evolution of the GO aggregates and the distribution of the director reaches saturation at 1.0 wt.%. At this concentration all of the liquid crystal is dominated by the GO percolation network. Also electro-optic properties and fractal analysis showed dramatic changes of the dispersions at this concentration^14^.

### Dielectric investigations

**Figure 4:**
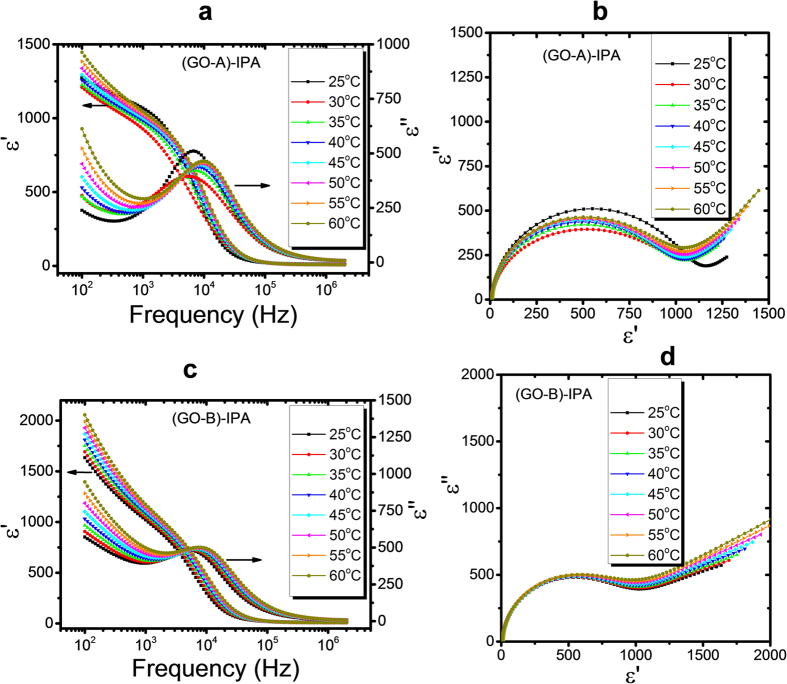
(**a**,**c**) Dispersion/absorption curves and (**b**), (d) corresponding Cole-Cole plots for the experimental data of GO-A in IPA (**a**) and (**b**), GO-B in IPA, (**c**,**d**) respectively.

Initially, dielectric spectroscopic measurements were carried out for different sizes of the GO-IPA suspensions, namely GO-A and GO-B, as a function of temperature. The real and imaginary part of the complex dielectric constant are depicted in Fig. 4(a,c), and the corresponding Cole-Cole plots are shown in Fig. 4(b,d). At low frequencies, the ionic contribution to the dielectric loss can clearly be seen. A complete relaxation mode is observed at frequencies of approximately 10 kHz, which can possibly be attributed to the GO flakes, most likely related to fluctuations of the normal of the graphene oxide plane, or in other words, rotations around axes in the plane of the GO flakes. The high dielectric constant has been related to the reorientation/rearrangement of the functional groups (-OH and -COOH) on the edge of the GO flakes^9^. In addition, the charge distribution on the graphene oxide surface could result in an electric dipole moment, increasing as the flake size increases. The asymmetry of the semi-circular curves (α ≠ 1) of the Cole-Cole plots can be explained by non-Debye behaviour^27^, i.e. dielectric relaxation processes from a combination of various mechanisms, or the size distribution of the GO flakes.

**Figure 5:**
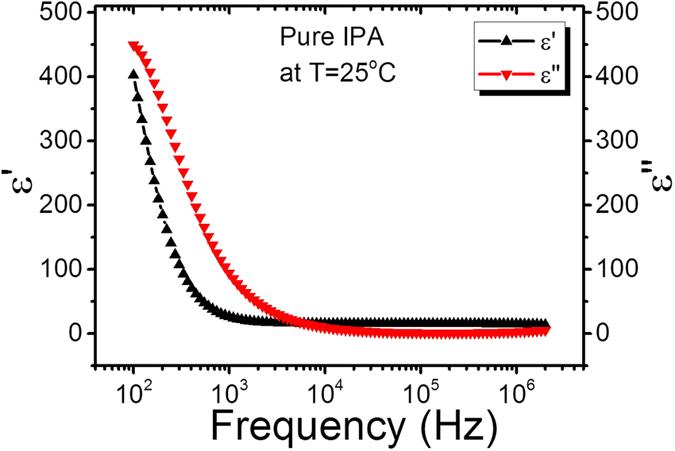
Dielectric dispersion/absorption curve of pure IPA at 25 °C.

The same experimental conditions as for the GO-IPA suspensions were employed to measure the dielectric permittivity of pure IPA without any graphene oxide dispersed. In this system, no dielectric relaxation peaks were observed at 10 KHz, (see Fig. 5). This confirms that the dielectric loss peak has to be attributed to the GO flakes.

**Figure 6:**
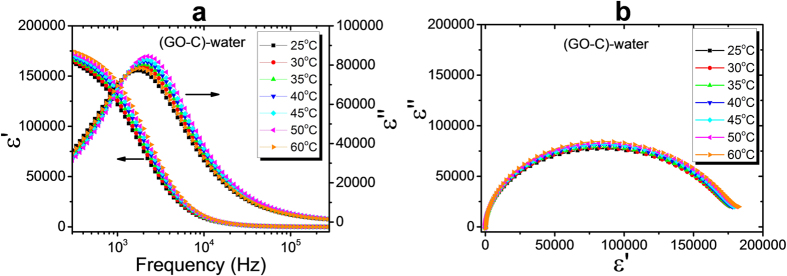
(**a**) Temperature-dependent dispersion/absorption curves for GO-C in deionized water and (**b**) corresponding Cole-Cole plots.

The dielectric properties of the largest GO flakes (GO-C) in deionized water were also investigated (Fig. 6). At this GO size range, flakes were well dispersible in water, forming a lyotropic nematic phase. Similar to the smaller flakes in IPA, also here a dielectric relaxation can be observed with relaxation frequencies in the order of 2 kHz, thus above ionic contributions, and slightly dependent on temperature. The apparently large polydispersity of sample GO-C does not seem to have a specific influence on the dielectric behaviour.

**Figure 7:**
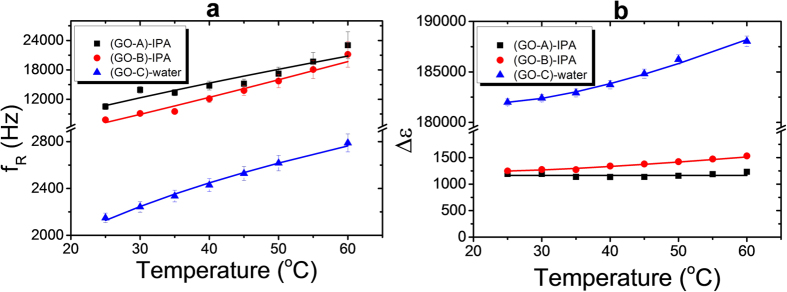
(**a**) Temperature dependence of the dielectric relaxation frequency f_R_ for graphene oxide flakes in isotropic media; (GO-A) and (GO-B) in IPA and (GO-C) in water. (**b**) Temperature dependence of the dielectric strength for the same materials as in (**a**).

Figure 7(a) shows the temperature dependence of the dielectric relaxation frequencies *f*_*R*_ to the GO flakes in the isotropic carrier fluids (IPA and water). Since the viscosity of the host fluid decreases with increasing temperature, the relaxation frequency of the graphene oxide increases with temperature, similar as it is observed for collective modes, like the Goldstone mode^28^. The relaxation frequency decreases for increasing graphene oxide flake size, due to the larger average moment of inertia for the rotational fluctuations. The dielectric strength Δε increases with increasing average size of the graphene oxide flakes, as shown in Fig. 7(b) and is practically constant over the temperature range under investigation. The differences between samples GO-A, GO-B and GO-C seem to be related more to the mean size of the graphene oxide flakes and the different solvent (IPA and water) than the polydispersity of the flakes.

**Figure 8:**
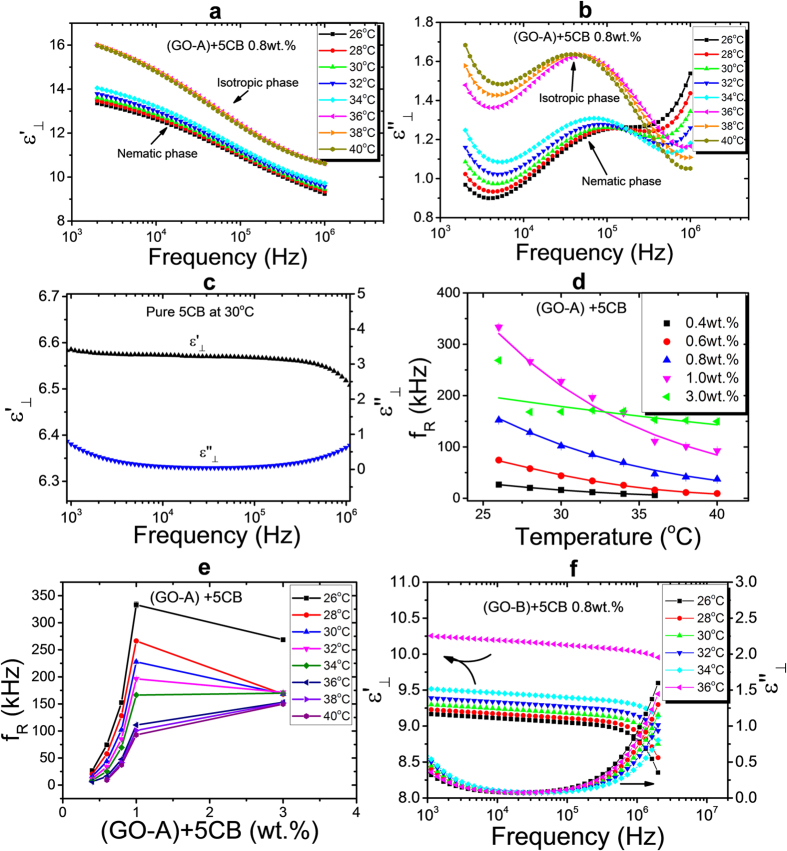
Frequency dependence of the perpendicular components of the dielectric constants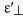 (**a**) and the dielectric loss 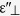 (**b**) of (GO-A) + 5CB 0.8% by weight. (**c**) 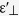 and 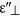 for pure 5CB. (**d**) The temperature dependence of the dielectric relaxation frequency f_R_ for different concentrations of (GO-A) in 5CB. (**e**) The dielectric relaxation frequencies as a function of (GO-A) concentration in 5CB at different temperatures. And (**f**) Dispersion/absorption curves of the dielectric constants perpendicular to the long molecular axis for (GO-B) in 5CB.

To gain further insight into the dielectric behaviour, the GO flakes (GO-A and GO-B) were additionally dispersed into the thermotropic nematic liquid crystal, 5CB. Since the medium is now anisotropic and possesses elastic properties, it can be expected that the dispersed graphene oxide would behave differently. Figure 8(a,b) show an example of the perpendicular components of the dielectric permittivity and loss of (GO-A) + 5CB at a concentration of 0.8 wt.% graphene oxide. A dielectric relaxation process can be observed at relatively high frequencies in the order of 50–100 kHz, which is not due to the so-called ‘cell mode’, as this behaviour is not observed for pure 5CB as a reference, nor due to the 5CB molecules, Fig. 8(c). This implies that the relaxations seen in the loss curves are due to the presence of the dispersed graphene oxide flakes. Furthermore, a clear discontinuity in dielectric strength and loss can be seen, as the transition from the doped nematic to isotropic phase is observed (Fig. 8(a,b), respectively).

Figure 8(d) depicts the temperature dependence of the dielectric relaxation frequencies *f*_*R*_ of the (GO-A) + 5CB suspensions for different concentrations. Contrary to the isotropic carrier media, here the relaxation frequency decreases with increasing temperature. Due to the competition of the temperature dependencies of the elastic constant and the viscosity, the GO-5CB system displays a more complex behaviour than the GO-IPA system.

Figure 8(e) illustrates the concentration dependence of the dielectric relaxation frequency f_R_ of the (GO-A)-5CB system. The critical changes in f_R_ are again observed at approximately 1.0 wt.%., which is the equivalent concentration where all of the liquid crystal is dominated by the dispersed graphene oxide, as has also been pointed out for other parameters of the mixtures determined in ref. 14. For increasing GO concentration, the aggregate or percolation network of the flakes becomes increasingly denser, with an increasing amount of bulk liquid crystal being dominated by the graphene oxide. At a threshold concentration of approximately 1 wt%, all of the liquid crystal is strongly influenced by the GO aggregate, and physical parameters saturate, such as viscosity, electro-optic response, or as shown in Fig. 8(e) the dielectric relaxation frequency.

Dielectric spectroscopy of the larger graphene oxide flakes (GO-B) + 5CB system was carried out under the same experimental conditions as above, and is shown in Fig. 8(f). In this system, however, no relaxation process is observed in the moderate frequency ranges. Thus, by comparing with the results of (GO-A) and (GO-B) doped 5CB, it can be concluded that the dielectric relaxation mode corresponding to the GO flakes can only be observed over a relatively narrow size range of GO if it is to be found at standard frequency ranges of 500 Hz-500 kHz. In addition, the smaller size ranges of GO can disperse better in the anisotropic liquid crystal medium, which results in increased space available to the flakes to behave as individual sheets.

**Figure 9:**
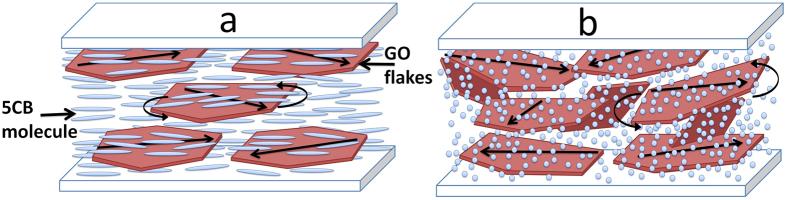
Schematic diagram of the liquid crystal cell filled with a mixture of GO flakes that are dispersed in (**a**) thermotropic liquid crystal medium (5CB), and (**b**) isotropic medium. The arrow on the flakes represents the direction of the electric dipole moment. In the presence of an electric field, the flakes could rotate by following the direction of the AC field.

Due to the difference in the physical properties of the isotropic and the anisotropic media, GO flakes are thought to react diffeently to the measuring electric field. For illustrative purposes, the behavior of GO in the liquid crystal cell is shown schematically in Fig. 9. The main reason that *f*_*R*_ in the nematic medium is 10 times larger than in the isotropic medium could be related to the elastic properties of the medium. The viscosity of 5CB is 81 mPa·s^17^, while that of isopropanol is 2.1 mPa·s. Thus in 5CB, the GO flakes have only limited freedom and fluctuate with an on avarage smaller angle. In the isotropic medium, having low viscosity, the flakes are able to fluctuate more freely.

Moreover, there are possibilities that the high relaxation frequency in the GO doped 5CB may be attributed to the slowing down of the molecular modes (rotation around short axis) of 5CB molecules due to the GO sheets. This would occur when the 5CB molecules were to respond freely in the bulk while being trapped and hindered by the GO aggregates, or being anchored through dispersion interactions on the surface of the GO flakes. Nevertheless, as experiments were carried out for different sizes, but at constant concentration of the two-dimensional graphene oxide sheets, size effects would not be expected in the latter scenario, but only for fluctuations of the GO flakes themselves. Also, there is no indication of a discontinuity in the relaxation frequency as the system changes from nematic to isotropic on heating. It is likely, that both effects, rotational fluctuations of the graphene oxide sheets and molecular dispersion interactions between GO and 5CB contribute to some extend to the relaxation behaviour observed for anisotropic host materials. Nevertheless, it is believed that the GO flake fluctuations play the main role.

### Arrhenius equation

The temperature dependence of the dielectric relaxation frequency, f_R_, can follow an Arrhenius relationship^29^,^30^


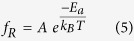


where *E*_*a*_ is the activation energy, T is the absolute temperature, k_B_ is the Boltzmann constant, and A is a pre-exponential factor.

**Figure 10:**
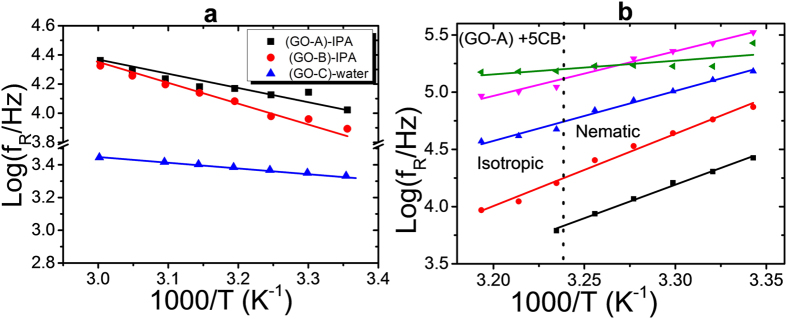
Logarithm of the dielectric relaxation frequencies as a function of inverse absolute temperature for GO in the isotropic media (**a**) and in the anisotropic medium (**b**).

The temperature dependence of the relaxation frequencies of the dielectric modes of the GO-isotropic suspensions, Fig. 10(a), follows a standard Arrhenius-like behaviour, while the relaxation frequencies of the GO doped 5CB, Fig. 10(b), show an opposite behaviour with the corresponding activation energies *E*_*a*_ being negative. Experimentally, the activation energies, *E*_*a*_, of the molecular relaxation modes of pure 5CB are found to be positive in both the nematic and the isotropic phases^31^,^32^. This indicates that the discrepancy in behaviour between the GO-isotropic and the GO-5CB suspensions is solely related to the GO flakes or their influence on the liquid crystal.

Negative activation energies *E*_*a*_ of the high relaxation frequency modes are not uncommon and have for example been found for polymer films of chitosan^33^. This has been explained by an activation process that involves the breaking of hydrogen bonds in their primary hydration shells, being followed by the formation of other hydrogen bonds. In the GO-5CB mixture, the hydrogen bonding between the hydrocarbon groups (-CH) of 5CB molecules and hydroxyle groups (–OH and -COOH) of GO flakes are more likely broken as a result of heating up. Nevertheless, in addition to possible hydrogen bonding, the mechanisms of the relaxation in the GO doped 5CB system, are more complex, because they also depend on the competition between the viscosity and the elastic constant of the anisotropic carrier fluid, and the response from the 5CB molecules.

Table 1 shows the activation energies of the GO-dispersion systems that are calculated from Fig. 10. The activation energy E_a_ in the GO-IPA systems increases from 16 to 24 kJ/mole, as the size of the GO is increased. This increase can be associated with the increased moment of inertia of the larger GO flakes, thus a reluctance to significant rotational fluctuations. However, due to the presence of elasticity in the GO-water system, which is in a nematic lyotropic liquid crystal state, the activation energy is smallest. In the GO doped thermotropic liquid crystal systems, the modulus of the activation energy decreases as the concentration of the GO is increased. This could be explained by the fact that the increase of the weight percentage of GO enhances the aggregation and thus reduces the bonding percentage between the GO flakes and 5CB molecules.

## Conclusion

Graphene oxide flakes of different size ranges were prepared and characterized by SEM. Systems of GO dispersed in isotropic carrier fluids, which lead to the formation of lyotropic nematic phases, as well as in thermotropic nematic liquid crystals were investigated by dielectric spectroscopy as a function of temperature. Dielectric relaxation processes for different sizes of graphene oxide flakes-dispersions could be identified, which are absent in the pure carrier fluids.

In the GO-isotropic systems, the relaxation frequencies depend on the size of the GO flakes; the smaller the flakes the faster is the relaxation frequency. The dielectric relaxation is thus attributed to rotational fluctuations of the graphene oxide sheets. By increasing the size of the flakes to about 10 μm, the dispersion started to exhibit lyotropic nematic phases, connected with a significant decrease of the relaxation frequency. This further indicates that there is a relaxation process that is solely linked to the fluctuation of the GO flakes in the isotropic carrier liquid. The temperature dependence of the relaxation frequencies showed an Arrhenius-type behavior with a positive activation energy. However, in the GO doped thermotropic liquid crystal (GO-5CB), the relaxation frequencies are only observed for small GO size ranges and for concentrations larger than approximately 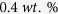. Furthermore, the temperature dependence exhibits an Arrhenius behavior with negative activation energies, which is discussed in terms of hydrogen bonding.

The ‘slow’ relaxation processes with frequencies in the order of (~10 kHz) for the GO-isotropic solvent system, can be related to rotational fluctuations of the graphene oxide planes. The faster relaxation processes with frequencies of about (~100 kHz) for the GO-nematic systems is more complicated and may be related to a combination of GO flake fluctuations and hindered mesogen rotation due to dispersion interactions between GO sheets and liquid crystal molecules.

## Additional Information

**How to cite this article**: Al-Zangana, S. *et al*. Dielectric spectroscopy of isotropic liquids and liquid crystal phases with dispersed graphene oxide. *Sci. Rep*. **6**, 31885; doi: 10.1038/srep31885 (2016).

## Figures and Tables

**Table 1:** Activation energies *E*_*a*_ of the GO-dispersions systems.

Sample	*E*_*a*_ (kJ/mole)
(GO-A)-IPA	16 ± 2
(GO-B)-IPA	24 ± 1
(GO-C)-water	6.2 ± 0.1
(GO-A) + 5CB (0.4 wt.%)	−112 ± 4
(GO-A) + 5CB (0.6 wt.%)	−121 ± 5
(GO-A) + 5CB (0.8 wt.%)	−84 ± 4
(GO-A) + 5CB (1.0 wt.%)	−75 ± 5
(GO-A) + 5CB (3.0 wt.%)	−23 ± 8
